# The Relationship Between Depressive Symptoms and Functional Gastrointestinal Disorders (FGIDs): The Chain Mediating Effect of Sleep Disorders and Somatic Symptom

**DOI:** 10.1155/2024/5586123

**Published:** 2024-11-20

**Authors:** Jiana Wang, Nana Meng, Kun Chen, Xinyuan Huang, Lin Feng, Cong Yang, Zhe Li, Xun Sun

**Affiliations:** ^1^School of Public Health, Health Science Center, Ningbo University, No. 818 Fenghua Road, Ningbo 315211, Zhejiang, China; ^2^Department of Social Medicine, School of Health Management, China Medical University, No. 77 Puhe Road, Shenyang, North New Area, Shenyang, Liaoning 110122, China; ^3^Anesthesiology Department, The Fourth Affiliated Hospital of China Medical University, Shenyang, Liaoning, China; ^4^Department of Immunology, Basic Medicine College, China Medical University, No. 77 Puhe Road, Shenyang, North New Area, Shenyang 110122, Liaoning, China

**Keywords:** chain mediation, depressive symptoms, FGIDs, sleep disorders, somatic symptoms

## Abstract

**Background:** More than two-thirds of patients with functional gastrointestinal disorders (FGIDs) experience various degrees of mental health issues. Although studies indicate that FGIDs are related to depressive symptoms, sleep disorders, and somatic symptoms, the underlying mechanism between these variables remains unknown. Our objective was to establish a model that outlines the interactions between these psychological dimensions in FGIDs and, thus, provide valuable insights into how to enhance the well-being of affected individuals.

**Methods:** This study used the convenient sampling method to enroll patients who visited the digestive internal medicine department. A total of 238 patients were investigated using the Rome IV criteria (irritable bowel syndrome used Rome Ⅲ criteria). A questionnaire including the Hospital Anxiety and Depressive Symptoms Scale, the Pittsburgh Sleep Quality Index, and the Patient Health Questionnaire-12 was used. The chain mediating roles of sleep disorders and somatic symptoms in the relationship between depressive symptoms and FGIDs were examined by the bootstrap method.

**Results:** Correlation analysis revealed that depressive symptoms were positively related to sleep disorders, somatic symptoms, and FGIDs. Sleep disorders were positively related to somatic symptoms and FGIDs. Somatic symptoms were positively related to FGIDs. Chain mediating effect analysis showed that depressive symptoms can not only affect FGIDs but also through three indirect paths, as follows: the mediating role of sleep disorders and somatic symptoms, the chain mediating roles of sleep disorders and somatic symptoms, and the mediating effect size accounted for 7.2%, 7.7%, and 2.5% of the total effect, respectively.

**Conclusions:** This study is conducive to understanding the internal mechanism underlying the relationship between depressive symptoms and FGIDs. It reminds us that when treating FGIDs patients, we should not only provide adequate psychological support to improve but also pay attention to improvements in their sleep quality and somatic symptoms.

## 1. Introduction

Functional gastrointestinal disorders (FGIDs) are a collection of chronic and recurrent gastrointestinal (GI) symptoms that occur without discernible organic changes in the GI tract [1]. A multinational study found that over 40% of individuals worldwide experience FGIDs [2]. In International Classification of Diseases 11th Revision (ICD-11), FGIDs are classified under digestive system diseases, emphasizing their importance in GI health [3]. Given their multifactorial origin and elusive etiology involving biological, psychological, and social factors [4], FGIDs lack a consensus on diagnostic biomarkers or curative therapies [5]. Currently, therapies often exhibit limitations [6] in terms of long-term efficacy and safety [7]. Importantly, FGIDs have been linked to a lower quality of life, increased doctor visits [1], and substantial healthcare costs [8], significantly impacting individuals' health-related quality of life and healthcare systems globally.

In recent years, psychological factors in FGIDs have received widespread attention, as the prevalence of psychological comorbidity in FGIDs is high [9]. Research shows that 54% of individuals with depressive disorders experience digestive discomfort symptoms [10]. The biopsychosocial medical model [11] requires us to abandon the linear causality of a single physiological cause. However, during initial consultations, patients typically present with predominantly physiological symptoms and discomfort. Although psychological factors play a significant role in the onset, development, and prognosis of FGIDs, unfortunately, the diagnostic criteria do not include psychological issues. Many nonpsychiatric practitioners often neglect these factors, resulting in excessive testing. Notably, patients with FGIDs often avoid attributing their condition to psychological factors [12].

Some research suggests that in FGID patients, psychological distress may significantly impact the quality of life to a greater extent than GI symptoms [13]. However, depressive symptoms, sleep disorders, and somatic symptoms, all play a role in FGIDs and their associated impaired quality of life, but their interplay remains poorly understood [14]. The association and underlying mechanisms of depressive symptoms and FGIDs are still unclear. Our objective is to explore a conceptual model outlining these psychological dimensions' interactions in FGIDs, aiming to establish a scientific basis for psychological treatments of FGIDs.

More than two-thirds of patients with FGIDs suffer from comorbidities, among which depressive symptoms are the most common [15]. Evidence suggests that depressive symptoms independently contribute to the development of FGIDs [16] and their severity correlates closely with GI symptoms [17]. The theory of social signal transduction [18] in depressive symptoms suggests that social stress heightens sensitivity to social pressures, leading to biological changes. Although some studies had proved that depressive symptoms could positively predict FGIDs, and preliminarily revealed the correlation between the two, there is still a lack of necessary empirical research on how depressive symptoms affect FGIDs.

To date, no research has investigated the possible mediating role of sleep disorders in the relationship between depressive symptoms and FGIDs. The stress-cognitive insomnia model [19] posits that depressive emotions may magnify occasional sleep disturbances, cultivating the belief of chronic poor sleep, thereby triggering emotional and physiological changes. Previous research established that depressive symptoms were significantly associated with sleep disorders [20], and that individuals with higher levels of depressive symptoms were more likely to suffer from sleep disorders [21]. Additionally, individuals with depressive symptoms may have elevated stress hormone levels, such as cortisol, further destabilizing sleep patterns [1].

A report demonstrated that poor sleep quality was associated with FGIDs [22], affecting hormone release through the hypothalamic–pituitary–adrenal axis and potentially exacerbating GI symptoms [23]. Sleep disturbances can weaken immune system function [24], trigger inflammatory responses, and affect neuroendocrine regulation [25], exacerbating FGIDs. Collectively, poor sleep quality and psychological stress are closely intertwined, potentially playing a contributory role in the onset of FGIDs [26]. However, research on the interplay between psychological factors, sleep quality, and FGIDs is lacking. We hypothesize that sleep disorders mediate the effect of depressive symptoms on FGIDs.

Somatic symptoms refer to the inclination to express psychological distress through physical and organic symptoms, often leading individuals to seek medical assistance [27]. In clinical practice, somatic symptoms are commonly used to explain many extraintestinal symptoms in patients with FGIDs, and they are also utilized to interpret coexisting symptoms of FGIDs [28]. Another study suggested that in patients with FGIDs, somatic symptoms were associated with the processes of GI sensation and movement [29]. Therefore, somatic symptoms are a robust risk factor for FGIDs [30].

Depressive symptoms and somatic symptoms may share underlying physiological and neurobiological mechanics [31]. About half of individuals with major depressive disorder report multiple unexplained somatic symptoms [32], indicating a strong association between the two. A study observed that psychological factors indirectly affect the severity of FGID symptoms mainly through somatic symptoms [33]. Unsurprisingly, the same psychological factors in our model, which predict the severity of FGIDs, also have predictive value for somatic symptoms. Therefore, we hypothesized that somatic symptoms mediate the effect of depressive symptoms on FGIDs.

Based on the reasoning above, the mediating roles played by sleep disorders and somatic symptoms have been given, but whether their role is parallel or chained needs further validation. Research has indicated that sleep disturbances are significant predictors of somatic complaints [34]. Individuals with sleep disorders exhibit heightened sensitivity to their bodily sensations, termed “body vigilance,” and tend to transform this vigilance into somatic symptoms [35]. Poor sleep quality can be a severe stress factor and can result in somatic effects [36]. In summary, we predicted that sleep disorders and somatic symptoms play a chain mediating role in the relationship between depressive symptoms and FGIDs.

In conclusion, depressive symptoms, sleep disorders, and somatic symptoms can all significantly predict FGIDs, and the relationships between them have been studied pairwise. However, the mechanism through which psychological factors cause FGIDs remains a significant challenge in the field of GI research. Based on the existing literature, few studies have explored placing these psychosocial factors in a theoretical model to investigate the mechanisms of their interactions. In light of this, we mainly aimed to test the mechanism through which depressive symptoms influence FGIDs, as follows: depressive symptoms are not only directly correlated with FGIDs but also indirectly affect FGIDs through the mediating effects of sleep disorders and somatic symptoms.

## 2. Methods

### 2.1. Study Design

This study was conducted at the Gastroenterology Department of a comprehensive hospital in Shenyang, Liaoning Province, China. The hospital serves a diverse population from the entire Northeast China area, ensuring a broad representation of patients in our study. In the outpatient process, patients first register. Then, the doctor conducts an interview and initial assessment to understand the patient's symptoms and medical history. If necessary, a routine physical examination is performed. Based on the assessment results, the doctor makes a diagnosis and prescribes medication, while also arranging for any follow-up tests and treatment plans. This study utilized the convenience sampling method to collect data from patients who visited the digestive internal medicine department in January and May 2023.

### 2.2. Participants

Overall, 238 questionnaires were distributed and recovered; 20 questionnaires were eliminated due to missing answers, while 218 valid questionnaires were obtained, for an effective response rate of 91.60%. The power analysis validated the adequacy of our sample size which demonstrates that with a sample size of 218, we have enough statistical power to detect mediation effects. All subjects with the following conditions were excluded according to the Rome IV criteria: (1) organic diseases that may affect GI symptoms include GI malignancies, gastric ulcers, esophagitis, GI infections, celiac disease, diabetes, and thyroid disease, etc.; (2) drugs that may cause GI dysfunction include calcium channel blockers and anticholinergic drugs, etc.; (3) surgery involving any part of the intestine except the appendix or gallbladder; and (4) patients were diagnosed with psychiatric disorders, underwent weight loss surgery, and were pregnant or lactating.

### 2.3. Ethics Statement

Statement that the study was performed in accordance with relevant guidelines and regulations. This research was approved by the Ethics Committee on Human Experimentation under project number XXX [2021] 78. Before the beginning of the data collection, each participant signed an informed consent form, which explained the purpose and content of the study and had the right to opt out of the study at any time. In this study, participation was completely anonymous, confidential, and voluntary.

### 2.4. Measurement

#### 2.4.1. Covariates

(1) Gender: Participants were asked to self-report their gender as either male or female. (2) Education background: Education levels were categorized into four groups based on self-reported highest educational attainment: “primary school and below,” “junior high school,” “high school,” and “college and above.” (3) Breakfast habits: Participants indicated their frequency of consuming breakfast as “never,” “sometimes,” or “often.” (4) Binge eating: Frequency of engaging in binge eating was categorized as “never,” “sometimes,” or “often.” (5) Dietary preference: Dietary habits were categorized into four groups based on reported preferences: “balanced nutritional combinations,” “primarily meat-based,” “mainly fruits and vegetables,” and “mainly focused on staple foods.” (6) Habit of drinking tea: Frequency of tea consumption was reported as “drink everyday,” “occasionally drink,” or “never drink.” (7) Drink milk in a week: Participants reported their weekly milk consumption as “never,” “occasionally,” or “frequently.” (8) Regular eating habits: Eating regularity was assessed based on participant responses indicating their eating habits as “irregular,” “occasionally irregular,” or “frequently irregular.” (9) Alcohol consumption: Frequency of alcohol consumption was categorized as “never,” “occasionally,” or “frequently.”

#### 2.4.2. Rome IV diagnostic questionnaire

According to the Rome IV criteria [37], FGIDs are classified into six major categories encompassing 33 diseases. Among them, only a few are notably common, including irritable bowel syndrome (IBS), functional constipation (FC), functional dyspepsia (FD), and others. Given the high prevalence and significant impact of these disorders on quality of life, these three disorders were included in our study. The diagnostic criteria refer to Rome Ⅳ and Rome Ⅲ [38]. Although the Rome IV diagnostic criteria were strictly followed, the “Chinese Expert Consensus on Irritable Bowel Syndrome (2015)” still recommends using the Rome III criteria for diagnosing IBS [39]. Consequently, IBS was diagnosed based on the Rome III criteria. Diagnosis of FD based on the Rome IV criteria requires the presence of one or more of the following symptoms for at least 6 months: bothersome postprandial fullness, early satiation, epigastric pain, and epigastric burning [37]. Diagnosis of FC based on the Rome IV criteria requires the presence of at least two of the following symptoms for at least 6 months: straining during defecation, hard or lumpy stools, a sense of incomplete evacuation, and a reduced frequency of stools [37]. To diagnose IBS according to the Rome Ⅲ criteria, patients must have had abdominal pain or discomfort for at least 3 of the previous 6 months, with two or more of the following symptoms: pain improvement after defecation, symptoms associated with a change in stool frequency, or symptoms associated with a change in stool form [38]. Rome III and Ⅳ criteria have been widely used among patients in China [40].

#### 2.4.3. Hospital Anxiety and Depressive Symptoms Scale (HADS)

The HADS [41] is a self-administered questionnaire commonly used by doctors to assess the levels of depressive symptoms experienced by individuals. The HADS comprises a total of 14 questions, with seven dedicated to depressive. Each question offers three possible responses, and the maximum score for the depressive subscale is 21. Scores ranging from 8 to 10, 11 to 14, and 15 to 21 indicate mild, moderate, and severe depressive symptoms, respectively. The Chinese version of HADS has been widely used in China and has adequate reliability and validity [42], and the Cronbach's alpha = 0.803.

#### 2.4.4. The Pittsburgh Sleep Quality Index (PSQI)

Sleep quality was assessed by the Chinese version of the PSQI, which was translated and validated by Liu and Tang [43]. The original scale was designed by Buysse et al. [44], which was used to measure the sleep quality and disturbances over the past month. It includes 18 items consisting of seven components: subjective quality of sleep, sleep latency, sleep duration, habitual sleep efficiency, sleep disturbance, use of sleep medication, and daytime dysfunction. Each item is scored from 0 (not during the past month) to 3 (three or more times a week), with the total score ranging from 0 to 21. A higher score suggests poorer sleep quality. A cutoff score of 5 was recommended for screening for sleep disturbance by Buysse et al. The Chinese version of PSQI has been widely used in China and has adequate reliability and validity [44]. In this study, the Cronbach's alpha = 0.716.

#### 2.4.5. The Patient Health Questionnaire-12 (PHQ-12)

The self-reported 12-item PHQ-12 [45] was used to assess somatic symptoms. The instrument is a modified version of the widely used PHQ-15 [46] that excluded three GI symptoms. The PHQ-12 includes a list of 12 symptoms (e.g., fatigue, pain) that account for more than 90% of the physical complaints reported in primary care. Each question has four response options: “not at all,” “several days,” “more than a week,” and “nearly every day.” This scale helps healthcare professionals gain a better understanding of the patient's physical condition, enabling them to develop more effective treatment plans. The questionnaire has been widely used in China [47]. In this study, the Cronbach's alpha = 0.86.

### 2.5. Procedure

The steps of the study included two parts: “doctor diagnosis” and “patient self-reports.” In the “doctor diagnosis” section, the doctor involved in the study was selected based on the Rome IV guidelines, possessing expertise in FGIDs using these standards and conducting psychosocial evaluations from a screening perspective. The gastroenterologist performed medical assessments directly in the outpatient department, adhering to the Rome IV standards (or Rome III standards for IBS). In the “patient self-reports” section, patients completed questionnaires with the assistance of trained survey personnel. This included explaining the questionnaire, ensuring informed consent, and helping patients answer questions accurately. Professional investigators supervised this process to ensure accurate informed consent and data transparency.

### 2.6. Data Analysis

All the data in this study were analyzed using Statistical Package for the Social Sciences (SPSS) Version 26.0 and the PROCESS program. The study variables were standardized. First of all, a power analysis using the Monte Carlo simulation method was conducted to validate the adequacy of our sample size. By performing 10,000 simulations, the results showed an estimated power of 0.82, indicating that our sample size is sufficient to reliably detect mediation effects. Descriptive analysis was also conducted to evaluate demographic characteristics and eating habits. We used Spearman correlation to analyze the relationships between depressive symptoms, sleep disorders, somatic symptoms, and FGIDs. The PROCESS program of mediation (Model 6) was used to perform a multiple mediation analysis to analyze the indirect effect of sleep disorders and somatic symptoms on the relationship between depressive symptoms and FGIDs. The model was adjusted for individual-level indicators including sex, education background, and eating habits (breakfast or not, engage in binge eating or not, dietary preference, habit of drinking tea, drink milk in a week, regular eating habits, and drinking alcohol). The bootstrap method was used for testing. Repeated samples were taken 5000 times, and 95% confidence intervals (CIs) were calculated. The indirect effect was considered significant when the 95% CI did not contain zero.

## 3. Results

### 3.1. Common Method Bias Test

Harman's single-factor test was used to assess common method bias [48]. Overall, 10 factors with eigenvalues greater than 1 were found; the variance explained by the first factor was 22.75%, which was less than the critical standard of 40%. Thus, common method bias was excluded from this study.

### 3.2. Descriptive Analysis

In total, 103 patients were diagnosed with FGIDs, accounting for 47.2%. The mean age of the sample (*n* = 218) was 39.81 years (standard deviation [SD] = 13.80); 48.24% (*n* = 41) of those samples were males.  Table 1 shows that people with primary school or lower education levels are more prone to FGIDs than those with higher education levels (*p*=0.039); people who never drink milk in a week (*p*=0.015) are more prone to FGIDs than those who drink milk. Another demographic information for the study sample can be found in Table 1.

### 3.3. Correlation Analysis of Depressive Symptoms, Sleep Disorder, Somatic Symptoms, and FGIDs

As presented in Table 2, four of the variables indicate positive correlations with one another (all *p* values < 0.01). For example, higher depressive symptoms scores (indicating higher level of depressive symptoms) are positively associated with higher levels of sleep disorders, higher level of somatic symptoms, and FGIDs (*r* = 0.251, 0.220, and 0.213, respectively).

### 3.4. Testing the Mediating Effects of Depressive Symptoms and FGIDs

**Figure 1:**
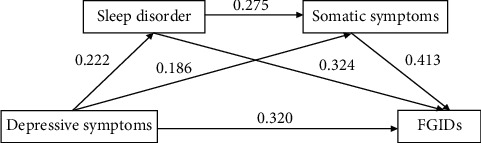
The chain mediating effect of sleep disorders and somatic symptoms between depressive symptoms and FGIDs. All coefficients are standardized coefficients; the solid line represents the path that is statistically significant (*p* < 0.05). FGIDs, functional gastrointestinal disorders.

Mediation analysis using the SPSS macroprogram process developed by Hayes was conducted to examine the mediating effects of sleep disorders and somatic symptoms on the relationship between depressive symptoms and FGIDs. Regression analysis revealed that depressive symptoms had a direct positive predictive effect on sleep disorders (*β* = 0.222, *p*  < 0.01) and somatic symptoms (*β* = 0.186, *p*  < 0.01). Sleep disorders had a positive predictive effect on somatic symptoms (*β* = 0.275, *p*  < 0.01). When simultaneously predicting FGIDs with depressive symptoms, sleep disorders, and somatic symptoms, all three factors exhibited positive predictive effects on FGIDs (*β* = 0.32, *p*  < 0.05; *β* = 0.324, *p*  < 0.05; *β* = 0.413, *p*  < 0.01). More details are displayed in Table 3 and Figure 1.

As presented in Table 4, the bootstrap method was used to test the mediating effects. The results showed that the indirect effect of sleep disorders as a mediator was 0.072 (95% CI = [0.004, 0.178]), with a bootstrap 95% CI not containing 0, signifying a significant mediating role of sleep disorders (depressive symptoms → sleep disorder → FGIDs). The indirect effect of somatic symptoms as a mediator was 0.077 (95% CI = [0.013, 0.164]), with a bootstrap 95% CI not including 0, indicating a significant chain mediating role of somatic symptoms (depressive symptoms → somatic symptoms → FGIDs). The indirect effect of depressive symptoms and somatic symptoms as mediators combined was 0.025 (95% CI = [0.004, 0.066]), with a bootstrap 95% CI not containing 0, demonstrating a significant chain mediating role of depressive symptoms and sleep disorders (depressive symptoms → sleep disorders → somatic symptoms → FGIDs). The total indirect effect of all mediating pathways was 0.174. The mediating effect of sleep disorders and somatic symptoms on the positive association between depressive symptoms and FGIDs was also supported.

## 4. Discussion

In this study, 103 patients, constituting 47.2% of the sample, were diagnosed with FGIDs. An epidemiological survey revealed that 40%–60% of patients with various subtypes of FGIDs were diagnosed with GI disorders in gastroenterology departments and clinics. Our research was consistent with this finding. The impact of educational level may reflect differences in health knowledge and lifestyle. Lower educational attainment is associated with limited health awareness and unhealthy lifestyle habits [49], thereby increasing the risk of FGIDs. The association between consuming milk and a reduced risk of FGIDs may be attributed to the presence of probiotics and other beneficial components in milk [50]. Milk is considered beneficial for intestinal health, and its role in maintaining a balanced gut microbiota may contribute to lowering the risk of FGIDs. This underscores the significance of dietary factors in GI health and offers new insights for potential nutritional interventions in the future.

This study revealed significant positive correlations between depressive symptoms, sleep disorders, somatic symptoms, and FGIDs, indicating that higher levels of depressive symptoms are associated with more severe sleep disturbances and somatic symptoms, and are more likely to be linked to FGIDs. These findings not only contribute to the understanding of the relationship between depressive symptoms and FGIDs but also provide a new perspective for the prevention and intervention of FGIDs.

### 4.1. Depressive Symptoms and FGIDs

The results of this study supported the hypothesis that depressive symptoms were positively associated with FGIDs. This finding suggested that higher levels of depressive are associated with more severe FGIDs, which is consistent with previous research and indicates the significant impact of depressive symptoms on the gut–brain axis [1]. When patients experience depressive, the digestion and emptying of the stomach can be significantly slowed [17]. With the advancements in neurogastroenterology and brain imaging, complex circuitry connections have been shown to exist between neural clusters involved in regulating higher order neural activities (such as emotions and cognition) in the central nervous system and those that modulate GI sensation and motility [51]. The possible mechanisms underlying this association are complex and multifactorial, involving psychological, physiological, social, and environmental factors. This finding suggests that treating depressive symptoms may help improve the FGID symptoms.

### 4.2. The Mediating Role of Sleep Disorders and Somatic Symptoms

Consistent with our hypothesis, we found that sleep disorders mediated the relationship between depressive symptoms and FGIDs. In other words, depressive symptoms can increase the severity of sleep disorders, thus leading to FGIDs. Therefore, sleep disorders are not only the result of depressive symptoms but also the cause of FGIDs. This result is consistent with previous research that sleep disorders are one of the root causes of FGIDs [22]. In addition, the results support the stress-cognitive insomnia model [19]. Specifically, stress triggers negative thoughts, creating a cycle that worsens insomnia. According to many previous studies, sleep disturbances comprise an independent disease and a risk factor for many other diseases, including GI disorders such as IBS, gastroesophageal reflux disease, gastric ulcers, and duodenal ulcer [52]. This means that poor sleep quality can cause or aggravate FGID symptoms by itself. This finding is in line with previous research revealing the biological mechanisms through which sleep disorders can affect the gut–brain axis [23].

Consistent with our hypothesis, somatization was found to be another important mediator. Depressive prompts somatic experiences such as pain, fatigue, or discomfort, which individuals might interpret as indicative of a physical illness. These symptoms can manifest in the GI tract due to its sensitivity to emotional states. Furthermore, the bidirectional communication between the gut and the brain, known as the gut–brain axis [4], plays a pivotal role. Altered gut microbiota composition and immune system responses, influenced by depressive, can contribute to the development and perpetuation of FGIDs. The somatization process serves as a bridge, linking the psychological distress of depressive to the physical realm and, subsequently, impacting the intricate balance of the GI system.

### 4.3. The Chain Mediation Model

This study revealed that depressive symptoms have a chain-mediated effect on FGIDs, through sleep disorders and somatic symptoms. Our findings were consistent with previous studies in which researchers found a positive correlation between sleep disorders and somatic symptoms. This relationship underscores that sleep disorders not only disrupt sleep patterns but also amplify individuals' awareness of bodily sensations. An impaired sleep quality might disturb the body's pain perception and emotional regulation systems, fostering a heightened somatic experience [53]. This heightened sensitivity to bodily sensations then contributes significantly to the manifestation of somatic symptoms.

With the increasing pressures of life, more and more people are finding themselves experiencing with symptoms of depressive. Research has shown that depressive not only leads to psychological distress but also leads to negative consequences, such as sleep disorders and somatic symptoms, etc. FGIDs occur as a result of the interaction of biological, psychological, and social factors in a biopsychosocial framework. Psychological distress is a well-established risk factor for FGIDs. The combined impact of depressive, sleep disorders, and somatic symptoms significantly heightened the risk of developing FGIDs in patients. The implications of these findings are important for both clinical practice and future research. In clinical practice, it suggests that improving sleep quality and reducing somatic symptoms may help improve FGID symptoms, especially for patients with depressive symptoms. Therefore, a comprehensive and holistic approach that addresses both the psychological and physical aspects of these disorders is recommended. For example, cognitive–behavioral therapy (CBT) can help FGID patients cope with their negative emotions and maladaptive cognitions related to their gut problems [54]. Future research should explore the differential effects of different psychological factors on sleep quality, somatic symptoms, and FGID symptoms, as well as the moderating and mediating factors that may influence these effects. For example, longitudinal studies can help establish the temporal sequence and causality of these variables.

## 5. Limitations

Several limitations should be considered when interpreting these findings. First, the cross-sectional nature of our data limits causal inference. However, theory-based mediation models supported by previous research can still provide valuable insights. Future studies should use longitudinal designs to test these models more robustly. Second, our reliance on participant self-reports could introduce recall bias. This study is a preliminary exploration. In future research, we plan to employ a longitudinal design and daily diary studies to better establish causal relationships and use more diverse data collection methods to accurately quantify FGIDs. Additionally, our study focused on the impact of depressive symptoms on FGIDs through sleep and somatic symptoms, but other psychological and relational variables may also play roles. Future research should include these variables and environmental factors for a more comprehensive understanding. Additionally, using convenience sampling may limit representativeness and introduce bias. Future studies should focus on increasing sample diversity and improving data collection methods to overcome this limitation. Lastly, while we adjusted for certain individual-level indicators (e.g., sex, education, eating habits), other potential confounders like socioeconomic status, medical history, and medication use were not fully addressed. These factors could have impacted our results.

## 6. Conclusions

This study identified the association between depressive symptoms and FGIDs as well as the mediating roles of sleep disorders and somatic symptoms. First, depressive symptoms can increase the risk of FGIDs by reducing sleep quality, and sleep disorders play a mediating role. Second, depressive symptoms can lead to somatic symptoms, subsequently causing GI disorders, and somatic symptoms play a mediating role. In addition, sleep disorders and somatic symptoms play chain mediating roles in the effect of FGIDs on depressive symptoms. These findings also suggested that FGIDs are systemic diseases involving not only the brain and the gut but also other systems and organs, such as the immune system, the endocrine system, and the cardiovascular system. Therefore, treating these diseases requires considering multiple factors, such as psychological, physiological, social, and environmental factors. Our findings provide promising directions for interventions to reduce the risk of FGIDs among people. Interventions should focus on reducing depressive symptoms and somatic symptoms and improving sleep quality. The findings of this study also serve as a reminder that individuals with depressive symptoms require comprehensive interventions that address not only their psychological well-being but also their somatic symptoms. Ranging from psychotherapy to pharmacological interventions, a holistic approach should be considered to achieve comprehensive health management.

## Figures and Tables

**Table 1:** Background and demographic information for overall sample.

Variables	*N* (%)	FGIDs		*χ* ^2^	*p*-Value
		Yes (%)	No (%)		
Gender					
Males	85 (38.99)	41 (48.24)	44 (51.76)	1.141	0.286
Females	133 (61.01)	59 (44.36)	74 (55.64)	—	—
Education background					
Primary school and below	8 (3.67)	3 (37.50)	5 (62.50)	8.351	0.039^*∗*^
Junior high school	33 (15.14)	12 (36.36)	21 (63.64)	—	—
High school	104 (47.71)	61 (58.65)	43 (41.35)	—	—
College and above	73 (33.49)	27 (36.99)	46 (63.01)	—	—
Breakfast or not					
Never	15 (6.88)	9 (60.00)	6 (40.00)	1.052	0.591
Sometimes	43 (19.72)	20 (46.51)	23 (53.49)	—	—
Often	160 (73.39)	74 (46.25)	86 (53.75)	—	—
Engage in binge eating or not					
Never	66 (30.28)	32 (48.48)	34 (51.52)	5.364	0.068
Sometimes	106 (48.62)	43 (40.57)	63 (59.43)	—	—
Often	46 (21.10)	28 (60.87)	18 (39.13)	—	—
Dietary preference					
Balanced nutritional combinations	65 (29.82)	29 (44.62)	36 (55.38)	5.243	0.155
Primarily meat-based	72 (33.03)	40 (55.56)	32 (44.44)	—	—
Mainly fruits and vegetables	26 (11.93)	14 (53.85)	12 (46.15)	—	—
Mainly focused on staple foods	55 (25.23)	20 (36.36)	35 (63.64)	—	—
Habit of drinking tea					
Drink everyday	31 (14.22)	18 (58.06)	13 (41.94)	2.066	0.356
Occasionally drink	151 (69.27)	67 (44.37)	84 (55.63)	—	—
Never drink	36 (16.51)	18 (50.00)	18 (50.00)	—	—
Drink milk in a week					
Never	94 (43.12)	55 (58.51)	39 (41.49)	8.416	0.015^*∗*^
Occasionally	78 (35.78)	30 (38.46)	48 (61.54)	—	—
Frequently	46 (21.10)	18 (39.13)	28 (60.87)	—	—
Regular eating habits					
Irregular	60 (27.52)	35 (58.33)	25 (41.67)	1.238	0.538
Occasionally irregular	117 (53.67)	59 (50.43)	58 (49.57)	—	—
Frequently irregular	41 (18.81)	19 (46.34)	22 (53.66)	—	—
Drink alcohol					
Never	59 (27.06)	25 (42.37)	34 (15.63)	4.083	0.13
Occasionally	121 (55.50)	55 (45.45)	66 (55.55)	—	—
Frequently	38 (17.43)	23 (60.53)	15 (39.47)	—	—

Abbreviation: FGIDs, functional gastrointestinal disorders.

*⁣* ^*∗*^ *p*  < 0.05.

**Table 2:** Means, standard deviations, and Pearson correlation.

Variable	Mean	SD	Depressive symptoms	Sleep disorders	Somatic symptoms
Depressive symptoms	8.91	3.87	—	—	—
Sleep disorders	4.77	2.72	0.251^*∗∗*^	—	—
Somatic symptoms	5.60	4.27	0.220^*∗∗*^	0.334^*∗∗*^	—
FGIDs	—	—	0.213^*∗*^	0.279^*∗∗*^	0.280^*∗∗*^

Abbreviations: FGIDs, functional gastrointestinal disorders; SD, standard deviation.

*⁣* ^*∗*^ *p*  < 0.05, *⁣*^*∗∗*^*p*  < 0.01.

**Table 3:** Regression analysis of the depressive symptoms, sleep disorder, somatic symptoms, and FGIDs regarding the chain intermediary model.

Regression equation		Global fit index			Regression coefficient significance	
Dependent variable	Independent variable	*R*	*R* ^2^	*F*	*β*	*t*
Sleep disorders	Depressive symptoms	0.222	0.049	11.172^*∗∗*^	0.222	3.342^*∗∗*^
Somatic symptoms	Depressive symptoms	0.364	0.133	16.43^*∗∗*^	0.186	2.849^*∗∗*^
	Sleep disorders	—	—	—	0.275	4.218^*∗∗*^
FGIDs	Depressive symptoms	—	—	—	0.320	2.096^*∗*^
	Sleep disorders	—	—	—	0.324	2.093^*∗*^
	Somatic symptoms	—	—	—	0.413	2.603^*∗∗*^

*Note:* Adjusted for sex, education background, and eating habits (breakfast or not, engage in binge eating or not, dietary preference, habit of drinking tea, drink milk in a week, regular eating habits, and drink alcohol).

Abbreviation: FGIDs, functional gastrointestinal disorders.

*⁣* ^*∗*^ *p*  < 0.05, *⁣*^*∗∗*^*p*  < 0.01.

**Table 4:** The mediating effect of sleep disorders and somatic symptoms on depressive symptoms and FGIDs.

Path	Standardized *β*	95% CI	
		Boot LLCI	Boot ULCI
Depressive symptoms → sleep disorders → FGIDs	0.072	0.004	0.178
Depressive symptoms → somatic symptoms → FGIDs	0.077	0.013	0.164
Depressive symptoms → sleep disorders → somatic symptoms → FGIDs	0.025	0.004	0.066
Indirect effects	0.174	—	
Total effect	0.320	—	

Abbreviations: CI, confidence interval; FGIDs, functional gastrointestinal disorders; LLCI, lower limits of the 95% confidence interval; ULCI, upper limit of the 95% confidence interval.

## Data Availability

The data are not publicly available because they contain information that could compromise research participant privacy and consent but are available from the corresponding author upon reasonable request.

## Nomenclature

FGIDs: Functional gastrointestinal disorders
IBS: Irritable bowel syndrome
FD: Functional dyspepsia
FC: Functional constipation
ICD-11: International Classification of Diseases 11th Revision
HADS: Hospital Anxiety and Depressive Symptoms Scale
PSQI: Pittsburgh Sleep Quality Index
PHQ-12: The Patient Health Questionnaire-12.
